# Coenzyme Q10 Ameliorates Ultraviolet B Irradiation Induced Cell Death Through Inhibition of Mitochondrial Intrinsic Cell Death Pathway

**DOI:** 10.3390/ijms12118302

**Published:** 2011-11-24

**Authors:** Li Jing, Santosh Kumari, Natalia Mendelev, P. Andy Li

**Affiliations:** 1 Department of Pharmaceutical Sciences, Biomanufacturing Research Institute and Technological Enterprise (BRITE), North Carolina Central University, Durham, NC 27707, USA; E-Mails: ljing@nccu.edu (L.J.); fkumari@NCCU.EDU (S.K.); nmendelev@nccu.edu (N.M.); 2 Department of Pathology, Ningxia Medical University, Yinchuan, Ningxia 750004, China

**Keywords:** apoptosis, caspase, cell death pathway, coenzyme Q10, mitochondria, ubiquinone 10, ultraviolet

## Abstract

Ultraviolet B (UVB) induces cell death by increasing free radical production, activating apoptotic cell death pathways and depolarizing mitochondrial membrane potential. Coenzyme Q10 (CoQ10), an essential cofactor in the mitochondrial electron transport chain, serves as a potent antioxidant in the mitochondria. The aim of the present study is to establish whether CoQ10 is capable of protecting neuronal cells against UVB-induced damage. Murine hippocampal HT22 cells were treated with 0.01, 0.1 or 1 μM of CoQ10 3 or 24 h prior to the cells being exposed to UVB irradiation. The CoQ10 concentrations were maintained during irradiation and 24 h post-UVB. Cell viability was assessed by counting viable cells and MTT conversion assay. Superoxide production and mitochondrial membrane potential were measured using fluorescent probes. Levels of cleaved caspase-9, caspase-3, and apoptosis-inducing factor (AIF) were detected using immunocytochemistry and Western blotting. The results showed that UVB irradiation decreased cell viability and such damaging effect was associated with increased superoxide production, mitochondrial depolarization, and activation of caspase-9 and caspase-3. Treatment with CoQ10 at three different concentrations started 24 h before UVB exposure significantly increased the cell viability. The protective effect of CoQ10 was associated with reduction in superoxide production, normalization of mitochondrial membrane potential and inhibition of caspase-9 and caspase-3 activation. It is concluded that the neuroprotective effect of CoQ10 results from inhibiting oxidative stress and blocking caspase-3 dependent cell death pathway.

## 1. Introduction

Ultraviolet (UV) spectrum has both beneficial and detrimental effects on human health. Although UVB (280 to 315 nm) represents only 4% of the total solar UV radiation, it is responsible for most of the medical conditions related to sun exposure. Increasing evidence indicates that the body response to UVB is a complex and multifaceted biological process. UVB triggers signal transduction at multiple intracellular sites and the crosstalk between dedicated molecular mediators acting within a complex signal network determines the fate of UVB irradiated cells. Even if little is known about the signaling mechanisms that are triggered by UVB in keratinocytes, it is well established that the detrimental effects of this type of irradiation are associated with the formation of reactive oxygen species (ROS) [1,2]. Several cytokines, growth factors, hormones, and neurotransmitters use ROS as secondary messengers in the intracellular signal transduction [3]. Conversely, at high concentrations, ROS are prone to cause damage and are thereby potentially toxic, mutagenic or carcinogenic due to their high reactivity [4,5]. All major groups of bio-molecules can be damaged by ROS-catalyzed reactions, undergoing structural and functional modifications. Although only retinal neurons among the neuronal cells have the chance to be exposed to UV light, UV irradiation has been employed as a useful model to study ROS-mediated pathophysiology in various cell types, including neurons. Our previous studies have shown that UVB induces cell death by increasing production of ROS, inducing mitochondrial membrane depolarization and activating mitochondria-initiated apoptotic cell death pathways that include activation of caspases-9 and -3 [6,7].

Coenzyme Q10 (CoQ10), a fat-soluble, vitamin-like benzoquinone compound, is the electron acceptor for complex I and II. CoQ10 is a potent antioxidant, a membrane stabilizer, and a cofactor in the production of adenosine triphosphate (ATP) by oxidative phosphorylation. CoQ10 is also known as ubiquinone-10 or ubiquinol-10, a crucial component in mitochondrial oxidative phosphorylation and ATP production [8]. CoQ10 is identified in the mitochondria, lysosomes, Golgi and plasma membranes, where it functions as an antioxidant either by direct reaction with free radicals or by regeneration of tocopherol and ascorbate from their oxidized state [9]. Loss of CoQ10 causes cardiac failure and mitochondrial defects [10]. Previous studies have shown that either intraocular or oral administration of CoQ10 or in combination with vitamin E minimizes the glutamate toxicity and protects retinal ganglion cells from retinal ischemia/reperfusion induced damage in rats [11]. CoQ10 ameliorates UVA induced damage in human keratinocytes through reduction of ROS accumulation and prevention of DNA damage [12]. The aim of this study was to define the mechanisms by which CoQ10 protects cells against UVB induced damage.

## 2. Results and Discussion

### 2.1. Effect of CoQ10 on UVB Irradiation Induced HT22 Cells Death

**Figure 1:**
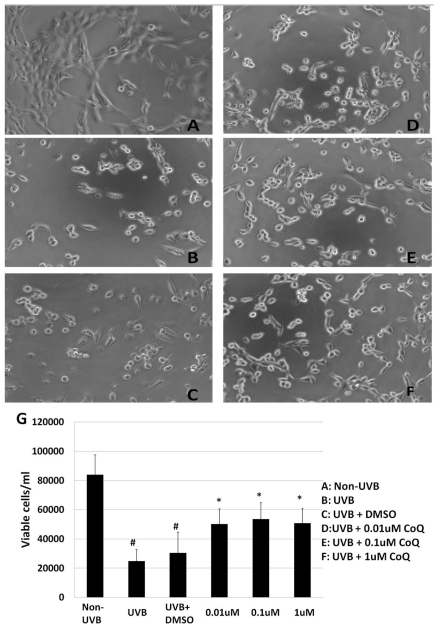
Cell viability after CoQ10 treatment in UVB exposed HT22 cells. UVB irradiation induced a significant reduction of cell viability (**B,C**) compared to control (**A**). Pretreatment with CoQ10 at 0.01, 0.1 or 1 μM improved cell viability at 24 h after UVB (**D–F**). Bar graph (**G**) represents 3 independent experiments, each performed in triplicate. # *p* < 0.01 *vs.* non-UVB control and * *p* < 0.05 *vs.* UVB. One-way ANOVA followed by Tukey’s test. Data are presented as means ± s.d.

To investigate the effect of CoQ10 on UVB-induced damage to HT22 cells, numbers of viable cells were counted in non-UVB-challenged (Figure 1A), UVB-challenged (Figure 1B), dimethyl sulfoxide (DMSO)-treated (Figure 1C), and CoQ10-treated HT22 cells (Figure 1D–F). UVB irradiation reduced the viable cells from 84,000 ± 13,456.62/mL to 24,800 ± 8043.63/mL, nearly a 75% reduction compared with non-UVB treated cells (*p* < 0.01). Addition of 0.1% DMSO did not influence of the cell survival rate after UVB-irradiation. Treatment with CoQ10 at a concentration of 0.01, 0.1 or 1 μM 24 h prior to UVB exposure and maintaining the same concentration for 24 h after UVB significantly increased the number of viable cells. As a result, at 24 h following UVB irradiation close to 50–60% of the HT22 cells was viable in CoQ10 treated cells (*p* < 0.05 *vs.* non-CoQ10 treated, UVB irradiated cells).

### 2.2. Influence of CoQ10 on Mitochondrial Succinate Dehydrogenase Activity

**Figure 2:**
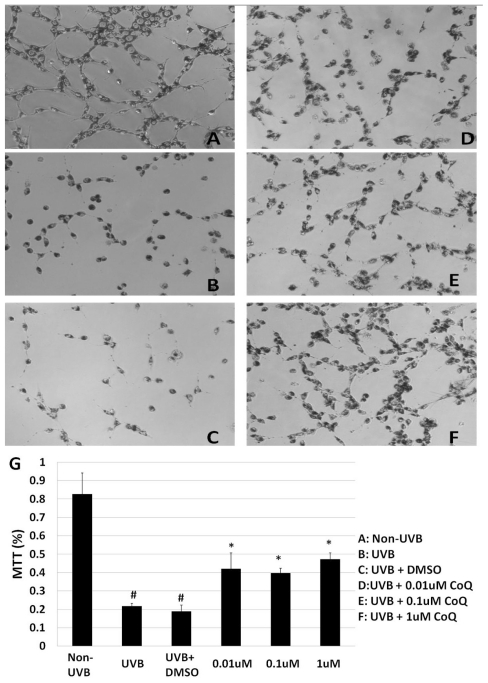
MTT assay. UVB irradiation suppressed mitochondrial succinate dehydrogenase activity (**B,C**) compared to control (**A**). Pretreatment with CoQ10 at 0.01, 0.1 or 1 μM improved mitochondrial enzyme activity at 24 h after UVB (**D–F**). Bar graph (**G**) represents 3 independent experiments, each performed in triplicate. # *p* < 0.01 *vs.* non- UVB control and * *p* < 0.05 *vs.* UVB. One-way ANOVA followed by Tukey’s test. Data are presented as means ± s.d.

The mitochondrial succinate dehydrogenase enzyme activity was detected using MTT assay. UVB-irradiation resulted in a significant decrease of MTT reduction, indicating a decrease of metabolically active mitochondria (Figure 2B) compared to Non-UVB control (Figure 2A). Addition of 0.1% DMSO did not protect HT22 cells from UVB induced damage (Figure 2C). Pre-treatment with CoQ10 initiated at 3 h prior to, and maintained for 24 h after, UVB irradiation, did not provide protection to mitochondria, as reflected by low conversion of MTT (data not shown). Similarly, MTT reduction remained as low as vehicle-treated cells if CoQ10 was only added at 24 h prior to UVB and discontinued after UVB exposure (data not shown). In contrast, CoQ10 pre-treatment initiated 24 h prior to, and maintained for 24 h after, UVB significantly intensified MTT reduction (Figure 2D–F). A summarized bar graph is given in (Figure 2G).

### 2.3. Effects of CoQ10 on ROS Production and Mitochondrial Membrane Potential

**Figure 3:**
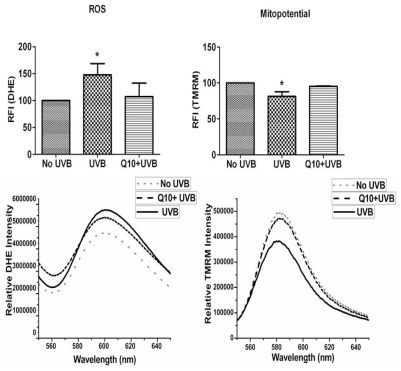
Measurements of ROS production by DHE (left column) and mitochondrial membrane potential by TMRM (right column). ROS bar graph demonstrates increases in ROS production after UVB and the anti-oxidative effect of CoQ10. Mitopotential bar graphs show mitochondrial membrane potential depolarization after UVB and preventive effect of CoQ10. Lower panels are representative original recordings. Data are collected form 3 independent experiments and presented as means ± s.d. * *p* < 0.05 against control. One-way ANOVA followed by Tukey’s test.

We measured ROS production using dihydroethidine (DHE) probe that detects superoxide. As shown in Figure 3 left panel, UVB irradiation significantly enhanced the production of ROS (*p* < 0.05) and pretreatment with 1 μM CoQ10 prevented the UVB-induced ROS increase. Thus the levels of ROS were the same in CoQ10 treated cells as in non-UVB irradiated controls (*p* > 0.05). Similarly, mitochondrial membrane potential (Figure 3 right panel), which was measured using tetramethylrhodamine methyl ester (TMRM), decreased in UVB-irradiated cells compared with normal control cells (*p* < 0.05), indicating mitochondrial membrane depolarization. Treatment with CoQ10 prevented the UVB-induced mitochondrial membrane depolarization. Therefore, the mitochondrial membrane potential in CoQ10-treated, UVB-irradiated cells was at the same level as in naïve control cells (*p* > 0.05).

### 2.4. Effect of CoQ10 on UVB Irradiation Induced Caspase-9 Activation

**Figure 4:**
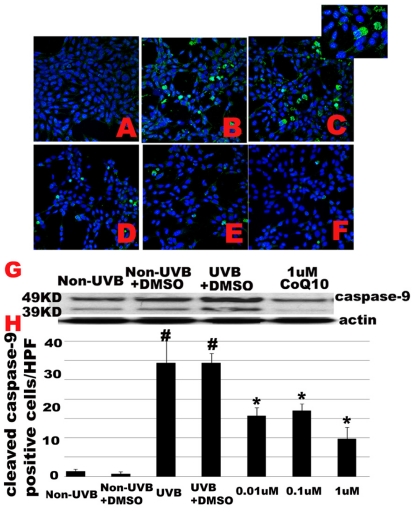
UVB-activated caspase-9 is inhibited by CoQ10. **A–F**, Immunocytochemistry of cleaved caspase-9. Number of caspase-9 positive cells increased after UVB irradiation with or without 0.1% DMSO (**B,C**) compared to control (**A**). Pretreatment with CoQ10 at 0.01, 0.1, or 1 M significantly reduced the number of caspase-9 positive cells (**D–F**). Green color represents cleaved caspase-9 staining and blue color denotes DAPI stained nuclei. Insert in Figure 3C shows cytosolic localization of cleaved caspase-9. **G**, western blotting showing full length (49 KD) and cleaved (39 KD) caspase-9 in control, UVB, and CoQ10 treated cells. Total and cleaved caspase-9 increased after UVB and CoQ10 pretreatment reduced both total and cleaved caspase-9 in the cytosolic fraction. **H**, Number of cleaved caspase-9 positive cells per high magnification field counted from 3 independent immunocytochemistry experiments, each performed in triplicate. # *p* < 0.01 *vs.* non-UVB control and * *p* < 0.05 *vs.* UVB. One-way ANOVA followed by Tukey’s test. Data are presented as means ± s.d. HPF, high magnification field.

Since CoQ10 is able to protect mitochondria, we decided to examine whether CoQ10 is capable of inhibiting mitochondria-initiated apoptotic cell death pathways that include activation of caspase-9 in the cytoplasm and nuclear translocation of apoptosis-inducing factor (AIF). Cleaved caspase-9 antibody was employed for immunocytochemisty and a set of representative photomicrograms is presented in (Figure 4A–F). No cleaved caspase-9 positive staining was observed in control cells without UVB challenge (Figure 4A). UVB-irradiation to cells cultured with normal medium (Figure 4B) or with addition of 0.1% DMSO (Figure 4C) induced a significant increased in caspase-9 positive cells (*p* < 0.01). The cleaved caspase-9 is localized in the cytoplasm (Figure 4C insert). Pretreatment with CoQ10 started 24 h prior to, and maintained for 24 h after, UVB (Figure 4D–F), significantly reduced the number of caspase-9 positively stained cells (*p* < 0.01) with no difference among the 3 dosages. Number of cleaved caspase-9 positively stained cells were counted per high magnification field (HPF) from 3 independent experiments, each performed in triplicate, and presented in (Figure 4H). Western blotting using caspase-9 antibody that detects both 49 KD full length caspase-9, and 39/37 KD cleaved caspase-9 further supports the findings observed in caspase-9 immunocytochemistry (Figure 4G). Thus, both the full length and a weak band of cleaved caspase-9 were observed in control cells cultured with or without 0.1% of DMSO. After UVB exposure, cleaved caspase-9 increased markedly. Pre-treatment with 1 μM Co-Q10 almost completely blocked the activation of caspase-9 (Figure 4G). Treatment with 0.01 μM and 0.1 μM achieved the same efficacy as 1 μM of Co10 on caspase-9 inhibition (data not shown).

AIF nuclear translocation was examined using anti-AIF antibody for immunocytochemistry. Although a few cells showed AIF positive staining in nuclei after UVB irradiation, such an increase did not reach statistical significance (data not shown). Number of AIF positively stained cells in CoQ10 treatment group was the same as UVB exposed cells (data not shown).

### 2.5. Effect of CoQ10 on UVB Irradiation Induced Caspase-3 Activation

**Figure 5:**
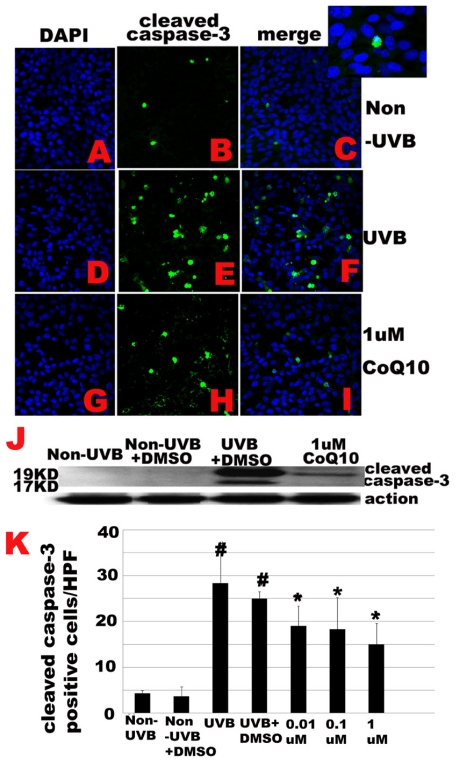
UVB-activated caspase-3 is inhibited by CoQ10. **A–F**, Immunocytochemistry of cleaved caspase-3. Number of caspase-3 positive cells increased after UVB irradiation (**D-F**) compared to control (**A–C**). Pretreatment with CoQ10 at 0.01, 0.1, or 1 M significantly reduced the number of caspase-3 positive cells (**G–I**). Green color represents cleaved caspase-3 staining and blue color denotes DAPI stained nuclei. Insert in Figure 5C shows nuclear localization of cleaved caspase-3. **J**, Western blotting showing cleaved caspase-3 in control, UVB, and CoQ10 treated cells. The cleaved caspase-3 increased after UVB and CoQ10 pretreatment reduced the cleaved caspase-3 in the nuclear fraction. **K**, Number of cleaved caspase-3 positive cells per high magnification field counted from 3 independent immunocytochemistry experiments, each performed in triplicate. # *p* < 0.01 *vs.* non-UVB control and * *p* < 0.05 *vs.* UVB. One-way ANOVA followed by Tukey’s test. Data are presented as means ± s.d.

Because cleaved caspase-9 activates caspase-3, the latter translocates into nucleus, causing DNA fragmentation and eventually cell death we decided to study whether CoQ10 blocks activation of caspase-3 in cells exposed to UVB. Cleaved caspase-3 (19/17 KD) was detected in UVB exposed cells treated with or without CoQ10 using immunocytochemistry and Western blotting. As shown in Figure 4, a few scattered caspase-3 positive cells were observed in control cells without UVB irradiation (Figure 5A–C). The location of the cleaved caspase-3 is in the nuclei (Figure 5C insert). The number of caspase-3 positive cells increased dramatically at 24 h following UVB irradiation, irrespective of DMSO was added or not (*p* < 0.01, Figure 5D–F). Treatment with CoQ10 (0.01, 0.1 or 1 μM) 24 h prior to, and maintained for 24 h after, UVB, significantly decreased the number of cleaved caspase-3 positive cells (*p* < 0.05, Figure 5G–I). Although high dose of CoQ10 (1 μM) intend to have better effect than the medium (0.1 μM) and low doses (0.01 μM), the differences did not reach statistical significance. Number of cleaved caspase-3 positively stained cells were counted per high magnification field from 3 independent immunocytochemistry experiments, each performed in triplicate, and presented in (Figure 5K). In agreement with the immunocytochemical finding, Western blot analysis using anti-caspase-3 antibody revealed that no cleaved caspase-3 in the nuclear fraction of the control non-UVB exposed cells with or without the presence of 0.1% DMSO in culture medium (Figure 5J). After UVB exposure, 19 and 17 KD cleaved caspase-3 increased markedly in the nuclear fraction and CoQ10 treatment initiated 24 h prior to, and maintained for 24 h after, UVB, significantly reduced the protein contents of activated caspase-3 (Figure 5J). The effect of CoQ10 on blocking caspase-3 activation was the same among all three tested dosages.

### 2.6. Discussion

Neuronal cells depend on mitochondrial oxidative phosphorylation for most of their energy needs. ROS are produced as by-products during mitochondrial electron transport, putting neurons at a risk for oxidative stress. Previous published studies, including ours, have demonstrated that UVB irradiation triggers neuronal HT22 cell death by enhancing ROS production, causing mitochondrial membrane potential depolarization, and activating mitochondria-initiated apoptotic cell death pathway [6,13]. The findings of the present study agree with previous published data showing that UVB irradiation reduces number of viable cells, inhibits the reduction of MTT, increases ROS production and causes mitochondrial membrane potential depolarization, suggesting that UVB induces cell death by affecting mitochondria. Further studies revealed that cleaved caspase-9 in the cytosolic fraction and cleaved caspase-3 in the nuclear fraction increased markedly after UVB exposure, implying UVB induced cell death is mediated through mitochondria-initiated apoptotic cell death pathway. A weak 39 KD cleaved caspase-9 band was observed in non-UVB exposed cells with or without DMSO. However, since there was no subsequent cleavage of caspase-3 observed, the meaning of the caspase-9 presented in non- UVB exposed cells is not clear. It is possible that the cleaved caspase-9 reflects a small number of cells undergoing apoptosis in baseline culture condition.

CoQ10 serves as both an electron transporter in the mitochondrial electron transport chain and a free radical scavenger. CoQ10 has been shown to attenuate UV-induced apoptosis, accumulation of ROS, and activation of caspase-3 in human cells harboring large-scale deletion of mitochondrial DNA [14]. In addition, prevention of apoptosis in keratinocytes by Co-Q10 is accompanied by inhibition of mitochondrial depolarization, cytochrome *c* release and caspase-9 activation, and such effects on mitochondria seems independent of CoQ10’s antioxidant property [15]. In the present study, we have shown that pretreatment with CoQ10 attenuated UVB-induced cell death. Thus, a partial response observed at 0.01 μM of CoQ10, which does not increase further with 1 μM of CoQ10. This suggests that the threshold for CoQ10 protection is below 0.01 μM. Future study has to use a dose range between 0.1–1 nM to satisfy the dose response curve fully. The protective effects of CoQ10 have been extensively studied in keratinocytes; however, its protective effects have not been well studied in neurons. In primarily cultured human keratinocytes collected from young and old donors, CoQ10 reduces UV-induced wrinkles by combating the age-related energy metabolism shifts that include a higher glucose uptake and lactate production [16], accelerating the production of laminin, a basement membrane component [17], and inhibiting the production of inflammatory cytokine IL-6 and matrix metalloproteinase 1 (MMP-1) [18]. The neuroprotective effects of CoQ10 have only been the focus of attention in the last 10 years. Studies from Dr. Pandy’s group in Canada have shown that water soluble CoQ10 protects hydroperoxide induced apoptosis in human teratocarcinoma NT2 cells and human neuroblastoma SH-SY5Y cells by reducing mitochondrial ROS generation, stabilizing mitochondrial membrane potential, reducing caspase-3 activity and attenuating the fall of ATP production [19]. In animal models of Parkinsonism induced by paraquat that is known to cause dopaminergic neurodegeneration, water soluble CoQ10 administrated prior to, or during, the paraquat treatment completely blocked the detrimental effects of paraquat on neuronal loss in the substantia nigra region and neurobehavioral deficit [20]. Similar results have also been observed in rotenone-induced Parkinsonism [21]. Diet supplementation of CoQ10 to aged transgenic mice with mutations in the amyloid precursor protein delayed brain atrophy defined by the volume change of the hemispheres and hippocampi measured using magnetic resonance image *in vivo* [22]. The molecular signaling pathways leading to neuroprotection of CoQ10 may involve inhibitions of BAX-induced mitochondrial dysfunction [23] and NFκB dependent pro-inflammatory gene expression [24]. In the present study, we demonstrate that CoQ10 protects neuronal cells from UVB-induced cell death by blocking activation of caspase-9 and -3, suggesting CoQ10 blocks mitochondria-initiated cell death pathway. It is not clear whether CoQ10 inhibits caspase-9 and -3 directly. We postulate that CoQ10 may inhibit the activation of caspase-9 and -3 by blocking upstream factors that cause the activation of these caspases. This is supported by our results showing that CoQ10 reduces ROS accumulation and prevents mitochondrial membrane depolarization in cells exposed to UVB. As is well known, mitochondrial depolarization opens mitochondrial permeability transition pore that allows release of certain mitochondrial proteins, causing activations of caspase-9 and, subsequently, caspase-3.

## 3. Material and Methods

### 3.1. Materials

Drugs and sources were as follows: Dulbecco’s modified Eagle’s medium (DMEM), phosphate buffered saline (PBS), Fetal bovine serum (FBS), Trypsin-Versene Mixture, L-Glutamine and Penicillin-Streptomycin solution were purchased from HyClone laboratories (USA). CoQ10, was purchased from Sigma-Aldrich (USA), dissolved in DMSO (Mediatech, USA) and diluted in culture medium.

### 3.2. Cell Culture, UVB Irradiation and CoQ10 Treatment

Murine hippocampal HT22 neuronal cells were cultured in DMEM containing 10% FBS, 2 mM glutamine, and 200 mM streptomycin/penicillin and then maintained at 90%–95% relative humidity in 5% CO_2_ at 37 °C. The culture medium was renewed every 3 days. Cells (80,000 to 15,000) were seeded in 12-well or 96-well cell culture plates and incubated in the above medium for at least 24 h in CO_2_ incubator to allow the cells reaching 80% confluence. Prior to UVB irradiation, the cultures were washed twice with PBS to remove residual serum and nonattached cells. Cells were incubated for 1 hour in serum-free medium and then exposed to 7 mJ/cm^2^ dose of UVB radiation with a Fisher UV Transilluminator FB-TI-88A. The UVB dose energy is calculated by UV intensity multiplying time of exposure. This dosage was chosen based on our previous study showing 50–70% cell death at 24 h post-exposure [13]. After UVB radiation, cells were returned to the culture incubator for 24 h of recovery at 37 °C. Three CoQ10 treatment protocols (each with concentrations of 0.01, 0.1 and 1 μM) were used: (1) CoQ10 was given 24 h prior to UVB and maintained during 3 min UVB; (2) CoQ10 was treated 24 h prior to UVB and maintained during UVB and 24 h post-UVB; (3) CoQ10 was given 3 h prior to UVB and maintained during UVB and 24 h post-UVB. 0.1% DMSO treated cells were used as control.

### 3.3. Cell Viability Assay

At 24 h after termination of UVB irradiation, numbers of viable cells in 12-well plates were counted using Beckman Coulter Vi-Cell Automated Cell Viability Analyzer (WS-VICELL), which uses trypan blue exclusion principle. The percent of cell viability was calculated and presented. All experiments were performed in triplicate and repeated in at least three separate experiments.

### 3.4. Mitochondrial Succinate Dehydrogenase Activity

Mitochondrial succinate dehydrogenase activity was measured in vehicle and CoQ10 treated murine hippocampal neuronal HT22 cells using 3-(4,5,-dimethythiazol-2-yl)-2,5-diphenyl tetrazolium bromide (MTT) conversion assay. Since conversion of yellow MTT to purple formazan requires succinate dehydrogenase and depends on the activity of the respiratory chain and state of redox mitochondria, MTT assay can be used to determine mitochondria activity, in addition to serve as a measure of cell viability. The cells were prepared for MTT staining after 24 h of recovery in 96-well plates following UVB injury. MTT (final concentration, 0.25 mg/mL) was added to the medium 2 h prior to pre-determined end-point and incubated for 4 h in 37 °C in dark. After incubation, cells were lysed in 50 μL DMSO that enables the release of dark purple reaction product, formazan. Absorbency at the wavelength 570 nm was read on an ELISA plate reader (Molecular Device, San Jose, USA). The results were expressed as a percentage of absorbency relative to control values.

### 3.5. Measurements of Superoxide and Mitochondrial Membrane Potential

Intracellular ROS (superoxide anion) production was measured using DHE probe in Co-Q10- pretreated cells exposed to UVB for 24 h. Briefly, cells (2 10^6^/mL) were incubated with the DHE (2.5 μM) for 30 min at 37 °C. Cells were washed, resuspended in phosphate buffered saline (PBS) and analyzed for fluorescence intensity using Fluoromax-4 spectroflorometer (HORIBA Jobin Yvon Inc, Edison, NJ) at the excitation and emission wavelengths of 480 nm and 590 nm respectively. The florescence recorded was represented as relative fluorescence intensity (RFI). Mitochondrial membrane potential was measured using the tetramethylrhodamine methyl ester (TMRM). Briefly, cells (1 × 10^6^/mL) were harvested and incubated with 30 nM TMRM at 37 °C for 1 h. Cells were washed in PBS and fluorescence measurement was performed with a Fluoromax-4 spectroflorometer (HORIBA Jobin Yvon Inc, Edison, NJ) at excitation and emission of 530 and 573 nm respectively. Mitochondrial potential was dissipated with carbonylcyanide p-trifluoromethoxyphenylhydrazone (FCCP 5 μM) and used as a positive control.

### 3.6. Western Blot Analysis

At 24 h following UVB treatment, cells were collected and lysed on ice in lysis buffer containing 20 mM Tris pH7.4, 10 mM KCl, 3 mM MgCl_2_, 0.5% NP40 and complete inhibitors (Millipore). Lysates were centrifuged at 500 *g* for 10 min and resulted in a supernatant (S1) and a pellet (P1). The S1 fraction was centrifuged at 20,000 *g* for 20 min and the resulting supernatant was used as a cytosolic fraction. The P1 fraction was washed twice with lysis buffer and resuspended in lysis buffer containing 1%SDS. The resulting lysates were sonicated briefly (Misonix, Ultrasonic Cell Disrupter) and then centrifuged at 20,800 *g* for 30 min. The resulting supernatants were designated as nuclear fractions. The purity of different cellular fractions has been tested previously [7]. Protein contents from each sample were measured using Microplate BCA Protein Assay Kit (Thermo Scientific). Equal amount of protein (20 μg) was loaded into each lane, separated in 10% NuPAGE BT gels (Invitrogen), transferred to PVDF membrane (Millipore), and probed with antibodies against AIF (D-20, Santa Cruz, 1;1000 dilution), caspase-3 (8G10, Cell Signaling, 1:1000 dilution) or caspase-9 (D353, Cell Signaling, 1:1000 dilution).

### 3.7. Immunocytochemistry

HT22 cells were fixed in 4% paraformaldehyde for 20 min at room temperature and processed for immunocytochemistry. After permeabilization with 0.1% Trinton X-100, the cells were incubated overnight at 4 °C with the polyclonal anti-AIF antibody (D-20, Santa Cruz, 1:300 dilution), monoclonal anti-cleaved caspase-3 antibody (ASP175,Cell Signaling:1:200 dilution) or polyclonal anti-cleave caspase-9 antibody (ASP353, Cell Signaling:1:50 dilution) followed by incubation with a secondary donkey anti-goat Alexa Fluor 488 (Invitrogen, Carlsbad, CA, USA) or donkey anti-rabbit Alexa Fluor 488 conjugate (Invitrogen) for 1 h at room temperature. The specimens were mounted with Vectashield Hardset Mounting Media (H-1200) containing DAPI and examined using a fluorescence confocal microscope (Nikon Eclipse C1). Three microscopic fields at 400X were captured and number of positively stained cells was counted.

### 3.8. Statistical Analysis

Data are presented as means ± s.d. One-way ANOVA followed by Tukey’s test was used for statistical analysis. A value of *p* < 0.05 was considered statistical significant.

## 4. Conclusion

Our results show that UVB irradiation induces cell death by targeting mitochondria and activating mitochondria-initiated cell death pathway involving caspase-9 and -3. Pretreatment with CoQ10 at 0.01, 0.1 or 1 μM initiated 24 h prior to, and maintained for 24 h after, UVB exposure, significantly increases cell viability by reducing ROS production, stabilizing mitochondrial membrane potential and blocking the activations of caspase-9 and caspase-3.
